# High mortality risk among women with acromegaly still persists

**DOI:** 10.3389/fendo.2024.1348972

**Published:** 2024-02-21

**Authors:** Simona Găloiu, Iustin-Daniel Toma, Denisa Isabella Tănasie, Andreea Bărbulescu, Ionela Baciu, Dan Alexandru Niculescu, Raluca Alexandra Trifănescu, Cristina Căpăţînă, Şerban Radian, Cătălina Poiană

**Affiliations:** ^1^ Department of Endocrinology, Carol Davila University of Medicine and Pharmacy, Bucharest, Romania; ^2^ Department of Endocrinology I, C. I. Parhon National Institute of Endocrinology, Bucharest, Romania

**Keywords:** acromegaly, mortality, pituitary tumor, somatostatin analogs, neurosurgery, pituitary radiotherapy

## Abstract

**Introduction:**

The mortality ratio in patients with acromegaly has improved over the last few decades. We aimed to determine the mortality rate and correlated factors in patients with acromegaly before and after the introduction of national protocols for treatment. In addition, we determined whether there are sex-related differences in mortality of patients with acromegaly.

**Methods:**

This observational retrospective study included 399 consecutive patients with acromegaly between January 2001–December 2022. Paraclinical data included random growth hormone (GH) and insulin-like growth factor-I (IGF1) levels, maximal pituitary tumor diameter at diagnosis, first visit, and last evaluation. Standardized mortality ratio (SMR) was calculated by dividing the observed and expected mortality rates. Cox regression analysis revealed the independent factors associated with mortality.

**Results:**

At the last visit, 31.07% (124) of patients were cured, 22.05% (88) had controlled acromegaly with medication, and 45.31% (181) had not controlled acromegaly. During follow-up (13.03 ± 5.65 years, 5216.62 person-years), 89 patients died (0.017%), resulting in an SMR of 1.18 [95% CI 0.95–1.45]. The independent factors associated with mortality were the last IGF1 level/last random GH level, absence of surgery, gonadotropin deficiency, and age. Patients with normal IGF1 after treatment showed an SMR of 0.71, whereas patients with IGF1 ratio > 1 showed SMR=1.51. Patients diagnosed between 1975–2007 and 2008–2022 had SMR = 1.25 [95% CI 0.97–1.58] and SMR = 1.09 [95% CI 0.68–1.65], respectively. In females with acromegaly, SMR was 1.63 [95% CI 1.24–2.11]; 1.76 [95% CI 1.30–2.34] in women diagnosed before 2008 and 1.33 [95% CI 0.69–2.33] in those diagnosed after 2008. Males with acromegaly had a mortality ratio similar to males from the general population (SMR = 0.99, [95% CI 0.66–1.41]).

**Conclusion:**

Patients diagnosed with acromegaly in the last 15 years had lower mortality rates than those diagnosed before 2008, due to the availability of new medications, primarily somatostatin receptor analogs and to a higher proportion of patients undergoing surgery. Females still have a high mortality ratio owing to older age at diagnosis and higher risk of metabolic complications. Therefore, efforts should be made for early diagnosis of acromegaly in women.

## 1 Introduction

Acromegaly is a rare disease that can lead to a shortened lifespan, if left untreated, owing to cardiovascular, metabolic, and cancer comorbidities. Earlier studies have shown that patients with untreated acromegaly have two–three times higher mortality rates than that of the general population (1, 2). However, with new therapeutic means available in the last few decades, more studies have reported a life expectancy in patients with controlled acromegaly similar to that of the general population. A pivotal study in 2005 reported normal standard mortality ratio (SMR) (1.16, 95% CI 0.85–1.54) for a national acromegaly cohort diagnosed between 1980 and 1999 (3). Notably, high mortality rate has been associated with basal serum growth hormone (GH) concentrations exceeding 2.5 ng/mL, a threshold proposed in numerous studies (2, 4, 5). Furthermore, Mercado et al. demonstrated a progressive decrease in survival of patients with GH levels increased from <1 ng/mL to >5 ng/mL (6).

Whether acromegaly treatment affects life expectancy has been questioned long ago; however, the impact of specific therapeutic modalities on mortality has not been sufficiently studied. Radiotherapy has been associated with increased mortality in patients with GH-secreting adenomas (7, 8), primarily by increasing cerebrovascular deaths, compared with those with nonfunctioning pituitary adenomas (9). Surgery and medical therapies are not directly linked to survival but act through normalizing GH and IGF1 levels. In a meta-analysis published in 2019, Bolfi et al. reported a pooled SMR of 0.98 in the subgroup of patients treated with somatostatin analogs (SSA) compared with an SMR of 2.11 in patients treated only with surgery and radiotherapy.

The interplay of sex differences in acromegaly remains a less-explored dimension, despite observations that women tend to be diagnosed at an older age than men (10, 11). According to a recently published study by Ritvonen et al. (12), females with acromegaly were older at diagnosis and death than males. Females with acromegaly had a higher mortality ratio than that of female controls. After 20 years of follow-up, the main cause of death shifted from cardiovascular to cancer in all patients. However, the absolute cancer rate in patients with acromegaly was not higher than that in the general population.

We aimed to determine the mortality rate and correlated factors predicting mortality in patients with acromegaly from a university referral center before and after the introduction of national protocols for treatment. In addition, we determined whether there are sex-related differences in mortality rates of patients with acromegaly.

## 2 Materials and methods

### 2.1 Patients

This observational retrospective study conducted in Department of Endocrinology I of the C. I. Parhon National Institute of Endocrinology, Romania, a tertiary referral endocrinology center included 399 consecutive patients diagnosed with acromegaly from all regions of the country (1000 estimated total number of patients with acromegaly diagnosed in Romania (13)) between 1975–2022 and admitted between 01.01.2001 and 31.12.2022. The inclusion criteria were as follows: age > 18 years, diagnosis of acromegaly, and follow-up for at least one year.

### 2.2 Treatment protocol

In Romania, the first therapeutic means for acromegaly in the 1990s included radiotherapy, neurosurgery, and dopamine agonist therapy. First-generation SSA treatment was introduced in 2006 and became valid for reimbursement claims and administered according to a national protocol since 2008, initially as a third-line therapy after surgery and radiotherapy. After GH receptor antagonists were introduced in 2010, medication became the second-line option for acromegaly, with radiotherapy as the third-line option. Patients with pituitary tumor diameters larger than 2 cm and without optic chiasma compression could have received SSA treatment without surgery (14). The second-generation SSA pasireotide was introduced in 2015.

### 2.3 Variables

Clinical data included the date of birth, date of acromegaly diagnosis, estimated duration of acromegaly symptoms, body mass index (BMI), systolic blood pressure, history of comorbidities such as diabetes mellitus, arterial hypertension, and dyslipidemia, type of treatment, date of acromegaly control, and date of death. Paraclinical data included the nadir GH in oral glucose tolerance test (OGTT), random GH and IGF1 levels, and IGF1 index, defined as the ratio of the measured value of IGF1 and upper normal limit for age and sex, maximal pituitary tumor diameter as determined by neuroimaging at diagnosis, the first visit (first evaluation after 2001), and the last evaluation.

Before 2008, serum GH levels were measured using an immunoradiometric method (sensitivity 0.1 ng/mL). Since 2008, serum GH levels have been measured using a chemiluminescence assay (Liaison, Sallugia, Italy), with a functional sensitivity 0.05 ng/mL. Serum IGF-I was measured using (generally measured various assays before 2012) Liaison IGF-I chemiluminescence assay (DiaSorin, Sallugia, Italy), with a sensitivity of 15 ng/mL.

### 2.4 Definitions

The diagnosis of acromegaly was based on the clinical features of excess GH and failure of GH suppression below 1 ng/mL in a 75 g OGTT and IGF1 > upper limit of normal (ULN) for age and sex. Acromegaly control was defined as random GH ≤ 1 ng/mL or nadir GH in OGTT ≤ 0.4 ng/mL and IGF1 ≤ ULN for age and sex (15).

Pituitary hormone deficiency was defined as follows: secondary hypothyroidism, as low free T4 level and low/normal TSH; hypogonadotropic hypogonadism, as amenorrhea/low estradiol in women or low testosterone in men, in the presence of low/normal follicle stimulating hormone and luteinizing hormone; and secondary adrenal failure, as serum 8 A.M. cortisol level below 5 µg/dL (insulin-induced hypoglycemia/is performed when the morning serum cortisol is between 5 and 15 µg/dL).

Arterial hypertension was defined as repeated office systolic blood pressure > 140 mmHg and/or diastolic blood pressure > 90 mmHg according to current guidelines (16). Diabetes mellitus was diagnosed according to the Standards of Medical Care in Diabetes-2022 edition (17): two values of fasting hyperglycemia > 126 mg/dL and/or glucose level > 200 mg/dL 120 min after a 75-g glucose load.

### 2.5 Statistical methods

According to their distribution, quantitative variables were presented as either mean ± standard deviation (SD) or median with interquartile range (IQR). Data distribution was determined using the Shapiro–Wilk test. Quantitative variables were analyzed using Student’s *t*-test or Mann–Whitney *U* test. For qualitative variables, we used either χ2 or Fisher’s exact tests. Mortality data for the Romanian population were obtained from the TEMPO-Online database of the National Institute of Statistics, Romania. Age- and sex-specific mortality rates of the general Romanian population were used to calculate the SMRs. Survival curves were plotted using Kaplan–Meier analysis, considering the time between the date of the first visit and the date of death or last follow-up. Cox proportional hazard analysis was used for multivariate analysis of competing risks. Statistical significance was set at *P* < 0.05. STATISTICA, (Data Science Workbench version 14; TIBCO Software Inc., 2020) was used as the statistical software (http://tibco.com).

## 3 Results

### 3.1 Characteristics of patients

A total of 399 patients with acromegaly, with a mean age at diagnosis of 44.32 ± 13.01 years, were included. The mean follow-up during the study was 13.03 ± 5.65 years, totalizing 5216.62 person-years.

Therapeutic means for these patients included neurosurgery in 65.66% (262) of the patients (primary by transsphenoidal approach (n=233) followed by transcranial surgery (n=29)), pituitary radiotherapy in 46.86% (187) of the patients, and medication in 63.90% (255) of the patients (163 patients received dopamine agonists, 188 patients received SSA, and 43 patients received GH receptor antagonists). Thirty-nine patients (9.52%) were evaluated only before any treatment. Twenty-eight non-operated patients received SSA. A total of 253 patients were diagnosed between 1975–2007 and 146 patients were diagnosed after 2008.

At the last visit, 31.07% (124) of patients were cured, 22.05% (88) had controlled acromegaly with medication, and 45.31% of patients (181) had not controlled acromegaly. The cure rate of surgery was 35.65%. Regarding cardiovascular and metabolic risk factors, 230 patients (57.64%) had arterial hypertension, 110 had diabetes mellitus (27.56%), and 213 (53.38%) had dyslipidemia. Pituitary failure on a minimum of one pituitary axis was noticed in 36.65% (145) patients, 125 (31.32%) had gonadotropin deficiency, 93 (23.30%) patients had thyrotropin deficiency, and 51 (12.78%) had corticotropin deficiency.

### 3.2 Mortality data

During follow-up (5216.62 person-years), 89 patients died (0.017%), compared to 75 expected, resulting in an SMR of 1.18 [95% CI = 0.95–1.45] for the entire patient cohort. Median age at death was 68.64 [59.36–75.02] years. Patients who died during follow-up were older at diagnosis than those who survived. Moreover, patients who died were less frequently treated surgically or with dopamine agonists, SSA, and/or GH receptor antagonists (**Table 1**). In patients who received SSA, the duration of treatment was shorter (1.70 [0.50–13] years) in those who died at the end of the study than in those who survived (4 [0.50–15.50] years). Radiotherapy was performed at a similar frequency in both subgroups of patients who survived and who died. An SMR of 1.17 [95% CI 0.86–1.55] was reported in patients who were irradiated.

**Table 1:** Comparison between deceased and alive acromegaly patients’ characteristics at the diagnosis and last visit.

Variable	Deceased	Alive	N	*P* (Mann–Whitney *U* test)
Age at diagnosis (years)	**48.81 [39.41–59.89]**	**42.42 [34.04–52.71]**	**395**	**0.0008**
Random GH at diagnosis (ng/mL)	10.40 [5.65–22.00]	12.80 [5.87–30.80]	342	0.286
IGF1 ratio at diagnosis (× ULN)	4.10 [2.58–4.60]	3.28 [2.30–4.06]	203	0.131
Maximal tumor diameter at diagnosis (mm)	**13.50 [8.00–19.00]**	**16.00 [11.00–23.00]**	**331**	**0.008**
Systolic blood pressure (mmHg)	**130.00 [120.00–150.00]**	**125.00 [115.00–135.00]**	**389**	**0.018**
Last GH (ng/mL)	**2.56 [0.80–13.80]**	**0.87 [0.36–3.00]**	**370**	**0.00001**
Last IGF1 ratio (× ULN)	**0.75 [0.88–2.62]**	**0.91 [0.70–1.24]**	**316**	**0.0003**
Last maximal tumor diameter (mm)	10.00 [6.50–17.00]	9.00 [5.50–13.00]	371	0.153
Surgery (proportion)	**33/89**	**229/310**	**399**	**0.00001**
Radiotherapy (proportion)	48/89	139/310	399	0.12
Dopamine agonist therapy (proportion)	**22/40**	**141/191**	**231**	**0.012**
Somatostatin analogue therapy (proportion)	**20/89**	**162/310**	**399**	**0.00001**
GH receptor antagonist therapy (proportion)	**1/89**	**42/310**	**399**	**0.0001**

Bold values are significantly statistically different.

At the last visit, higher values of systolic blood pressure, percentage of patients with arterial hypertension, percentage of patients with gonadotropin deficiency, and the last value of random GH and IGF1 index were observed in patients who did not survive compared with patients who were alive at the end of the study.

The independent factors associated with mortality revealed by the Cox survival analysis were the last IGF1 level, absence of surgery, gonadotropin deficiency, and age (**Table 2**). When the last random GH level replaced the last IGF1 level, independent factors correlated with mortality were the last GH (HR=1.01, 95% CI 1.004–1.018, *P* = 0.0008), absence of surgical treatment, and age.

**Table 2:** Factors correlated with mortality (Cox regression analysis).

Variable	HR	95% CI	*P*
**Last available IGF1 ratio**	**1.63**	**1.36–1.95**	**< 0.0001**
Gender (female vs. male)	0.89	0.46–1.72	0.72
**Surgery (no vs. yes)**	**1.97**	**1.05–3.11**	**0.03**
Radiotherapy (no vs. yes)	1.53	0.82–2.87	0.17
Somatostatin analogue therapy (no vs. yes)	1.00	0.51–1.97	0.98
**Gonadotropin deficiency (yes vs. no)**	**3.18**	**1.43–7.07**	**0.003**
Corticotropin deficiency (no vs. yes)	0.59	0.27–1.28	0.18
**Age at first visit (years)**	**1.10**	**1.07–1.14**	**< 0.0001**

Bold values are significantly statistically different.

**Figure 1:**
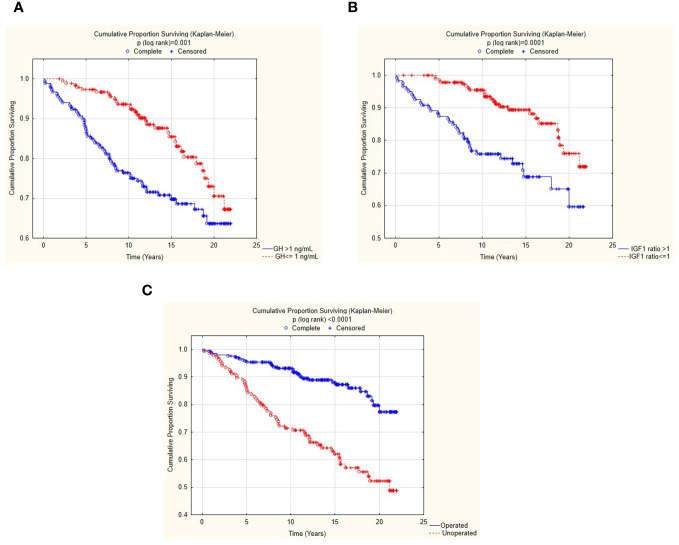
Kaplan–Meier survival curves in patients with random GH > 1 vs. ≤ 1 **(A)**, IGF1 index > 1 vs. ≤ 1 **(B)**, and receiving vs. not receiving surgical treatment **(C)**.

Patients with normal IGF1 after treatment (last IGF1 ratio ≤ 1) had SMR of 0.71, whereas patients with IGF1 ratio > 1 had 51% higher mortality rate than expected with SMR of 1.51. Similarly, patients with last GH ≤ 1 ng/mL had mortality comparable with the general population (SMR = 0.89), whereas patients with last GH > 1 ng/mL had SMR of 1.55. Kaplan–Meier analysis confirmed the impact of the last GH and IGF-1 indices on mortality (**Figures 1A–C**).

In the subgroup of 63 patients with the last GH level between 1-2.5 ng/ml, an SMR of 1.10 [95% CI 0.59–1.89] was observed. On the other hand, patients with IGF1 levels between 1 and 1.2 x ULN (n=57) had an SMR of 0.79 [95% CI 0.36–1.50].

Patients diagnosed between 1975–2007 had an increased mortality ratio by 25% with an SMR of 1.25 [95% CI 0.97–1.58] compared with that of the general population. Patients diagnosed between 2008–2022 had a mortality ratio similar to that of the general population, with an SMR of 1.09 [95% CI 0.68–1.65]. Regarding treatment efficacy during the two periods, of 245 patients diagnosed before 2008 at the final visit, 50.61% were cured after surgery and/or radiotherapy, 13.46% had controlled acromegaly with medication, and 35.91% had uncontrolled acromegaly. Of 146 patients diagnosed after the beginning of 2008, 38.35% were cured (50.90% (56/110) after surgery), 37.6% had controlled acromegaly, and 23.7% had not controlled acromegaly.

### 3.3 Sex-related differences in pituitary tumor characteristics and mortality rates

Females were older at diagnosis than males (45.72 ± 12.37 years vs. 41.59 ± 13.82 years, respectively, *P* = 0.002); they had smaller pituitary tumors (15 [10–21] mm vs. 17 [13–24] mm, respectively, *P* = 0.01), lower random GH (7.4 [2.90–18.60] ng/mL vs. 10.70 [4.93–23.30] ng/mL, respectively, *P* = 0.005), and lower IGF1 index (3.00 [2.21–3.80]vs. 3.81 [3.27–4.74], respectively, *P* = 0.00008) than those of males. Moreover, females had higher HbA1c levels than that of males (**Table 3**), whereas the frequency of diabetes mellitus was similar in females and males (*P* = 0.58). There were no significant sex-related differences in pituitary tumor characteristics at the last visit (GH, IGF1 ratio, and pituitary tumor maximal diameter) and systolic blood pressure. Males had more frequent pituitary failure on gonadotropin (41.66%) and thyrotropin (30.07%) axes than females (26.55% and 20.07%, respectively; *P* < 0.0001 and 0.006, respectively). However, higher number of males than that of females with gonadotropin deficiency received sex hormone steroid treatment (35/55 vs. 14/70; *P* < 0.0001).

**Table 3:** Comparison between characteristics of males and females with acromegaly at the diagnosis and final visit.

Patients characteristics	Male (n=135)	Female (n=264)	*P*
Follow-up (years)	13.33 [8.25–16.83]	13.16 [9.41–18.25]	0.51
**Age at diagnosis (years)**	**41.59 ± 13.82**	**45.72 ± 12.37**	**0.001**
Age at death (years)	69.40 [59.36–74.77]	67.35 [58.61–75.26]	0.87
Systolic blood pressure (mmHg)	130.0 [120.0–140.0]	121.0 [118.0–140.0]	0.48
Glucose (mg/dL)	97.00 [88.0–108.0]	96.40 [86.00–109.0]	0.63
**HbA1c (%)**	**5.70 [5.30–6.00]**	**5.90 [5.50–6.60]**	**0.004**
**GH at diagnostic (ng/mL)**	**15.00 [8.00–35.70]**	**10.35 [5.15–24.10]**	**0.005**
**IGF1 ratio at diagnosis (× ULN)**	**3.81 [3.27–4.74]**	**3.00 [2.12–3.80]**	**< 0.0001**
**Maximal tumor diameter (mm) at diagnosis**	**17.00 [13.00–24.00]**	**15.00 [10.00–21.00]**	**0.03**
Last GH (ng/mL)	1.10 [0.36–9.20]	1.01 [0.42–3.30]	0.73
**Last IGF1 ratio (× ULN)**	**1.07 [0.74–1.76]**	**0.90 [0.67–1.20]**	**0.008**
**Last maximal tumor diameter (mm)**	**10.00 [6.70–15.60]**	**8.30 [5.50–12.20]**	**0.01**

Bold values are significantly statistically different.

Further, a significant higher mortality rate was observed in females with acromegaly (59 actual vs. 36 expected), compared with matched women from the general population: SMR was 1.63 [95% CI 1.24–2.11]. Survival frequency was similar between both sexes (*P* (log-rank test) = 0.8) and as well as the age at death.

Females diagnosed with acromegaly before 2008 had a significantly increased mortality rate of 76% with SMR of 1.76 [95% CI 1.30–2.34]. Females diagnosed after 2008 had a decrease in SMR to 1.33 [95% CI 0.69–2.33]. Males with acromegaly had a mortality ratio similar to males from the general population with SMR of 0.99 [95% CI 0.66–1.41] (diagnosed before 2008: SMR = 1.007 [95% CI 0.63–1.52] and diagnosed after 2008: SMR = 1.07 [95% CI 0.51–1.98]).

## 4 Discussion

Mortality rates have improved over the last two decades (18–20). Mercado et al. reported a successful reduction in mortality rates in a retrospective study of 442 patients with acromegaly (65% females) to an SMR of 0.72 (6). In addition, a recent cohort study of 262 patients with acromegaly diagnosed between 1999–2019 in Norway demonstrated a decreasing trend of risk of mortality from 0.88 in patients diagnosed between 1999–2005 to 0.86 in those diagnosed between 2006–2012 and 0.52 in 2013–2019 (20).

Our study found a decrease in mortality rates of patients diagnosed with acromegaly between 1975–2022 with a median follow-up period of 13 years. Patients diagnosed after 2008 had similar mortality rates to the general population, whereas those diagnosed before 2008 had a 25% higher mortality rate than expected. Post-therapy levels of GH/IGF1, surgical therapy, and age were independent factors correlated with mortality. Patients with uncontrolled acromegaly, i.e., GH > 1 ng/mL (SMR = 1.55) and IGF1 ratio > 1 (SMR = 1.51), had a 50% increased mortality risk compared with the general population. Values of GH or IGF1 that are close to the upper limit of normal, i.e. between 1 and 2.5 ng/ml for GH and between 1 and 1.2 times the upper limit of normal (ULN) for IGF1, have been linked to mortality rates similar to those of the general population. Patients with IGF1 levels between 1 and 1.2 times the ULN have a better prognosis. Therefore, the 1.2 x ULN limit for IGF1 can be used as a target for therapeutic efficacy.

The absence of surgical therapy was identified as a significant independent factor that correlated with mortality. However, although neurosurgery is the first therapeutic option for acromegaly, only 65% of our cohort underwent pituitary surgery, including patients diagnosed before 2008, the period when radiotherapy was commonly used. Further, 28 patients diagnosed after 2008 with a pituitary tumor diameter > 2 cm received medication for acromegaly without neurosurgery due to the low probability of cure, according to the National Therapeutic Protocol for Acromegaly published in 2010 (14).

The cure rate of surgery was 35.65% at our center. The Belgian registry (21) had a similar proportion of patients who underwent surgery; the study reported cure rates of 34% for surgery and 34% for surgery and radiotherapy. Radiotherapy alone normalized GH and IGF1 in 50% of 20 patients. Biermasz et al. (22) observed a control rate of 37% ten years after surgery, whereas in the AcroBel registry, the global cure rate was 49%. Contrary to our results, radiotherapy was significantly associated with mortality in that study, with an SMR of 2.70 in irradiated patients, confirming the results of studies by Ayuk and Ritvonen (7, 12). In our study, patients treated with radiation did not have an increased risk of death, which is similar to the findings of the study by Mercado (6).

Colao et al. reported that partial surgical removal of GH-secreting pituitary tumors (> 75% of the tumor volume) significantly improved the response to SSA administered both before and/or after surgery (23). Another study of 73 consecutive patients with acromegaly from our center showed significantly lower GH and IGF-1 levels in patients treated with SSA after pituitary surgery than in the primary SSA treatment group, in which none of the patients was optimally controlled (24). Although not curative, our present study supported the role of surgery in patients with acromegaly independent of GH and IGF1 levels, as demonstrated by a significantly higher mortality rate in non-operated patients than in those who had benefited from the operation.

The GH control rate with multimodal therapy in patients with acromegaly at our center was published in 2020, with 77% of patients having normal GH levels among those evaluated between 2012 and 2019, and 46.7% having both normal GH and IGF1 (25). The first report of mortality in patients with acromegaly from our center, followed for a median of 7.35 years, showed an SMR of 1.34 (95% CI 0.96–1.83), while patients with the last random GH > 1 ng/mL had SMR of 1.59 (95% CI 1.08–2.26). Females with the last GH level > 1 ng/mL had a double mortality ratio compared with females in the general population (26). These novel findings support previous observations and show an improvement in the SMR to 1.33 in women diagnosed with acromegaly after 2008.

The effects of sex on acromegaly have been debated. An even sex distribution was observed in a Danish cohort of 569 patients with acromegaly and in a meta-analysis of 33 studies. Only a few papers from Bulgaria, Serbia, and Spain had a female preponderance of more than 60% of patients with acromegaly (27). In this meta-analysis, female patients were older (had a mean weighted age at diagnosis of 47.0 years for females and 43.6 years for males) and experienced a longer diagnostic delay. One possible explanation could be that the milder phenotype of acromegaly in females could pass without recognition, which could lead to prolonged exposure to excess GH and a greater risk of diabetes. A female predominance was also observed among our patients. They had milder tumor characteristics at the diagnosis of acromegaly, with lower GH, IGF1 index, and pituitary tumor diameters, and at the final visit, better IGF1 index and pituitary tumor diameters than those in males. However, females with acromegaly showed a higher mortality of 63% than that of matched females from the general population, whereas males with acromegaly had survival equal to the general population. Older age at diagnosis and metabolic complications due to prolonged exposure to excess GH may have contributed to high mortality rates in females. These findings are in line with those of Mercado et al., who found diabetes mellitus to be more common in females and significantly associated with baseline GH level > 10 ng/mL (6, 12).

A large study on two European cohorts of patients with hypopituitarism due to nonfunctioning pituitary adenoma found that gonadotropin deficiency conferred the highest relative risk for death of all pituitary axis (SMR-2.85) and that this risk was normalized with hormone replacement in men. This association could not be demonstrated in women because of the small proportion of women treated with estrogens (20%) (28). Our study confirmed that gonadotropin deficiency in patients with acromegaly, which starts at the reproductive age, is another factor associated with mortality. Additionally, the lack of steroid hormone substitution therapy, which was more common in women, may have contributed to the higher mortality rate among females.

The particularities of our large cohort of patients with acromegaly from the same neuroendocrine department are the high proportion of females and the therapeutic protocol. We used radiotherapy in a higher proportion of patients than in other centers, probably due to the low cure rate of surgery, even after the introduction of medical treatment. However, lowering GH/IGF1 levels with medication, primarily SSA, which has been used in most patients treated after 2008, and the debulking effect of neurosurgery and radiotherapy improved the mortality rate in all patients with controlled acromegaly. Radiotherapy did not increase mortality risk in our study.

## 5 Conclusions

Patients diagnosed with acromegaly in the last 15 years had lower mortality rates than those diagnosed before 2008. This better prognosis is mainly due to the introduction of new medications, mainly SSA, which efficiently lowered GH/IGF1 levels, and to a higher proportion of patients undergoing surgery due to changes in treatment protocols. Females still have a high mortality ratio owing to older age at diagnosis and a higher risk of metabolic complications. Therefore, efforts should be made for early diagnosis of acromegaly in women.

## Data availability statement

The raw data supporting the conclusions of this article will be made available by the authors, without undue reservation.

## Ethics statement

The studies involving humans were approved by Ethics Committee, C. I. Parhon National Institute of Endocrinology, Bucharest, Romania. The studies were conducted in accordance with the local legislation and institutional requirements. The participants provided their written informed consent to participate in this study.

## Author contributions

SG: Conceptualization, Formal analysis, Investigation, Methodology, Writing – original draft, Writing – review & editing. I-DT: Data curation, Investigation, Writing – original draft. DT: Data curation, Investigation, Writing – original draft. AB: Data curation, Investigation, Writing – original draft. IB: Writing – review & editing. DN: Writing – review & editing. RT: Writing – review & editing, Writing – original draft. CC: Writing – review & editing. ŞR: Writing – review & editing. CP: Writing – review & editing, Formal analysis, Project administration.
